# A Rare Case of Carcinoma in the Thyroglossal Duct Cyst of an Elderly Patient

**DOI:** 10.7759/cureus.1365

**Published:** 2017-06-19

**Authors:** Sundaramurthi Sudharsanan, Chellappa Vijayakumar, Srinivasan Dharanya, TP Elamurugan, Ali S Manwar

**Affiliations:** 1 Surgery, Jawaharlal Institute of Postgraduate Medical Education and Research (JIPMER), Puducherry, India.; 2 Otorhinolaryngology, Jawaharlal Institute of Postgraduate Medical Education and Research (JIPMER), Puducherry, India.

**Keywords:** thyroglossal duct cyst, papillary carcinoma, sistrunk operation, total thyroidectomy, elderly

## Abstract

Thyroglossal duct cysts are the most common congenital abnormalities of the neck, constituting about 70% of all cervical neck masses in children and 7% of the adult population. The occurrence of carcinoma in a thyroglossal duct cyst is very rare (less than 1%). Malignancy of the thyroglossal duct cyst usually presents in the third or fourth decade of life. We report a case of carcinoma in the thyroglossal duct cyst, which presented for the first time in our elderly patient.

A 76-year-old male presented with a 6 x 5 cm swelling in the anterior aspect of the neck. The swelling had been present for three months and had a variegated consistency. It moved with deglutition as well as with protrusion of the tongue. Intra-operatively, the lesion was cystic and was adherent to the hyoid bone. Sistrunk operation was done. The post-operative histopathology showed papillary carcinoma in the thyroglossal duct cyst.

The standard treatment is Sistrunk procedure with close follow-up of the patient. Patients with metastatic disease require a total thyroidectomy and in the presence of neck secondaries, neck dissection has to be done.

The diagnosis can be missed because of the rarity of this condition. Carcinoma should be suspected in a thyroglossal duct cyst when presenting for the first time in the elderly age group.

## Introduction

Thyroglossal duct cysts are the most common congenital anomaly of the thyroid gland. They are the second most common cause of neck masses in adults (7%), next only to lymph node swellings. Malignancies of thyroglossal duct cysts are very rare and constitute about 0.7% to 1% of all the thyroglossal cyst cases. They usually present in the third decade of life, commonly in women, with a male: female ratio of 1:1.5 [1]. Papillary carcinoma is the most common histological type and carries a good prognosis. The standard treatment is Sistrunk operation, with the addition of total thyroidectomy in high-risk cases. We report a rare case of papillary carcinoma in the thyroglossal duct cyst which presented for the first time in our patient at 76 years of age.

## Case presentation

**Figure 1:**
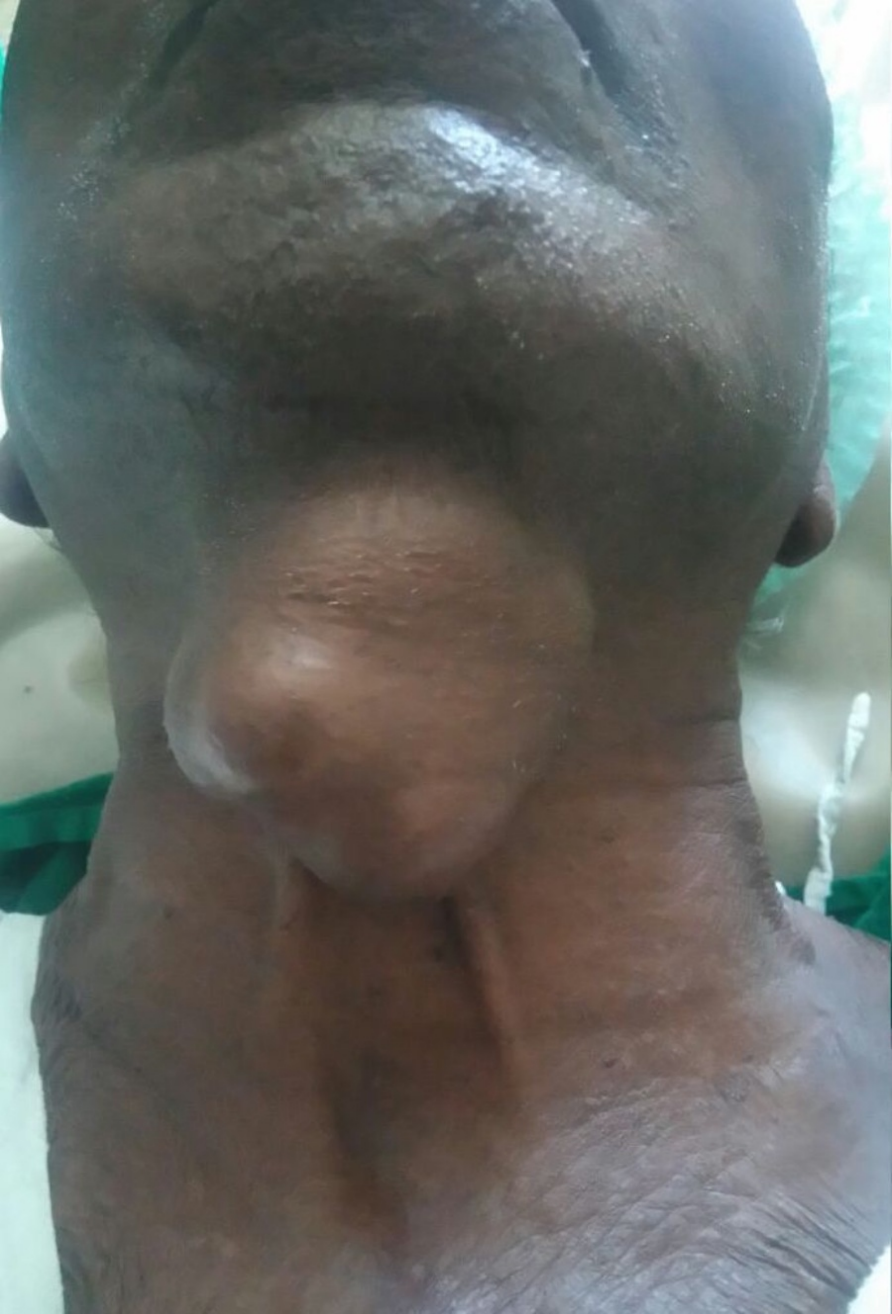
Clinical picture showing a 6 x 5 cm lobulated mass in the anterior aspect of the neck.

A 76-year-old male patient with no known co-morbidities presented with a swelling in front of the neck. The swelling was present for three months. It was gradually progressing in size and was associated with occasional pain. There were no significant pressure effects associated with the swelling. There were no symptoms suggestive of hypothyroidism or hyperthyroidism. On examination, there was a 6 x 5 cm swelling in front of the neck anterior to the thyroid cartilage, which was moving with deglutition and protrusion of the tongue (Figure 1).

**Figure 2:**
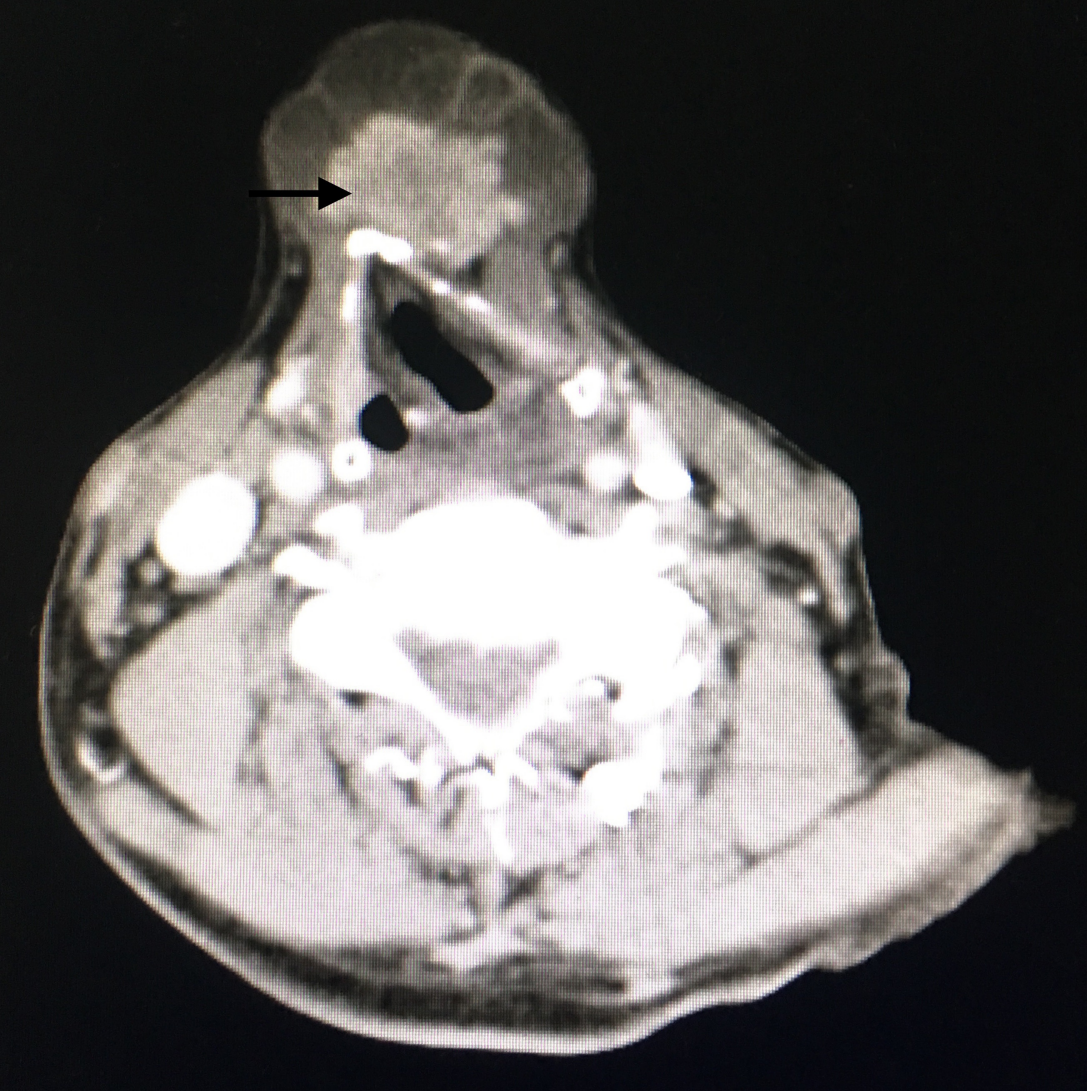
Contrast-enhanced CT scan showing a multiseptated cystic lesion with enhancing mural nodule anterior to the thyroid cartilage (arrow).

The swelling was non-tender and had variegated consistency with some parts being cystic and others firm. Clinically, a diagnosis of the thyroglossal duct cyst (with a possible malignant change) was made. A computed tomography (CT) scan of the neck was taken which showed a well-defined heterogenous cystic lesion of 5 x 4.5 cm with multiple enhancing internal septations noted in the sub-hyoid region. A homogenously enhancing component of size 3 x 1.8 cm was noted within the above lesion (Figure 2).

The normal thyroid gland was visualized in the usual position; there was no significant lymph node enlargement. The swelling was noted to be abutting the hyoid and thyroid cartilage with no obvious erosions. An ultrasound-guided fine needle aspiration cytology (FNAC) was done from the nodule within the swelling, which showed an atypia of undetermined significance. The thyroid function tests were found to be within normal limits.

The patient was taken for surgery. Intra-operatively, a 5 x 5 cm cystic swelling with solid components was found. The swelling was adherent to the hyoid bone, and a part of the hyoid bone was excised along with the entire thyroglossal tract. Two suspicious lymph nodes were isolated and sent for frozen section examination. 

**Figure 3:**
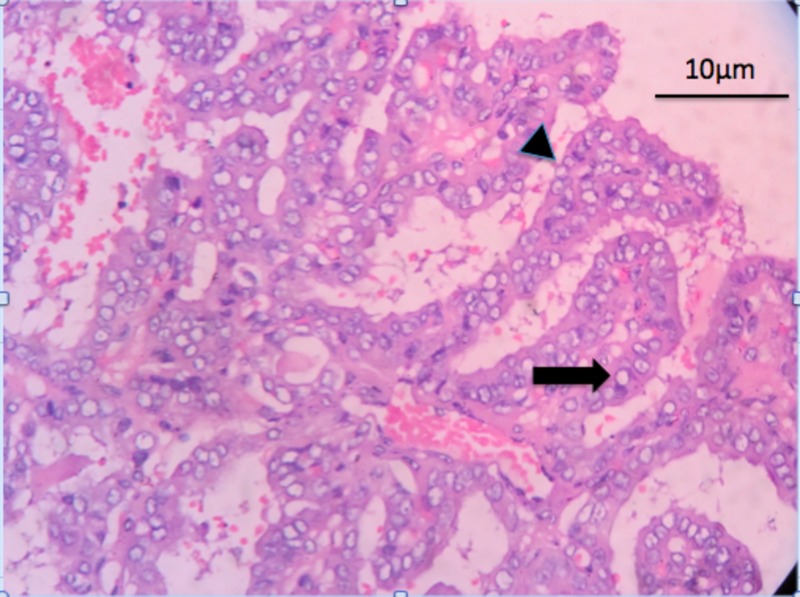
Hematoxylin and eosin (H&E) staining showing complex papillary architecture (arrowhead) with a fibrovascular core. The tumor cells have nuclear clearing - Orphan Annie eye nuclei (arrow).

Microscopic examination showed complex branching papillae with tumor cells having abundant eosinophilic cytoplasm, nuclear overlapping, nuclear cleaving and grooving, and the characteristic Orphan Annie eye nuclei with eccentric indistinct nucleolus (Figure 3).

Mitotic figures were frequent (2-3 / high-power field). Hemosiderin laden macrophages and foreign-body type giant cells were seen in the stroma. No normal thyroid parenchyma was seen. Both the lymph nodes were free of the tumor. A diagnosis of papillary carcinoma arising in the thyroglossal duct cyst was made.

## Discussion

The thyroid gland descends down from the foramen caecum, in front of the hyoid bone and the laryngeal cartilages, and reaches its final position in front of the trachea by the seventh week of gestation. During the migration, the thyroid gland remains connected to the foramen caecum at the base of the tongue by a narrow epithelial tract, named the thyroglossal duct. Usually, the duct obliterates within 8 to 10 weeks of gestation. A failure of complete involution of the thyroglossal duct results in the development of a thyroglossal duct cyst. 

Thyroglossal duct cyst is the most common congenital anomaly of the thyroid seen in around 7% of the adult population. They constitute about 70% of midline neck masses in children [2]. Thyroglossal cysts can be seen anywhere from the base of the tongue to the thyroid cartilage; more than 50% of the thyroglossal duct cysts are seen close to or just inferior to the body of the hyoid bone. They are benign lesions and usually present as midline cystic neck swellings that move with deglutition as well as with the protrusion of the tongue. Ectopic thyroid tissues can be seen in some of these cysts and such swellings can present as solid masses.

Brentano first described carcinoma in a thyroglossal cyst in 1911 [3]. They are very rare with a reported incidence of 0.7% to 1% of all thyroglossal cysts [1]. Carcinoma usually arises from the ectopic thyroid tissues within the thyroglossal cyst. The most common malignancy in a thyroglossal duct cyst is the papillary carcinoma (90%) followed by the squamous cell carcinoma (5%) [4]. Other malignancies like follicular carcinoma, Hurtle cell neoplasm, and mixed papillary-follicular carcinomas have also been reported in a thyroglossal duct cyst. Squamous cell carcinoma is considered the true carcinoma of the thyroglossal duct cyst as they develop from the cyst epithelium, whereas the other malignant histological types develop from the ectopic thyroid tissue present in these cysts [5]. 

The development of malignancy in a thyroglossal duct cyst is explained by two theories, the de novo theory and the metastatic theory. According to the de novo theory (the most accepted theory), malignancy develops from the ectopic thyroid tissue present in these cysts. This is also supported by the fact that medullary carcinomas are never seen to arise from a thyroglossal duct cyst, as the cells of origin are parafollicular cells, which are not seen in the ectopic thyroid tissue in the thyroglossal cysts. According to the metastatic theory, the thyroglossal duct cyst carcinoma is considered to be a metastasis from an occult primary carcinoma of the thyroid. Joseph and Komorowski proposed strict criteria for the diagnosis of thyroglossal duct cyst carcinomas that includes thyroglossal remnant, the presence of ectopic thyroid tissue within the thyroglossal cyst, and the presence of a normal thyroid gland.

Clinically, it is difficult to differentiate between a benign and a malignant thyroglossal duct cyst. Local invasion is seen in up to 4% of malignant cases where the swelling becomes fixed to the surrounding structures, and cervical lymph node secondaries are seen in 7% to 11% of the cases. Distant metastases are seen in only 2% of the cases of papillary carcinoma arising in a thyroglossal duct cyst [6]. 

Ultrasonography is the initial diagnostic modality of choice of midline neck swellings. The radiologic findings of malignancy include the presence of a mural nodule within a cystic lesion, micro-calcifications, and the presence of enlarged cervical lymph nodes. CT scans can evaluate the infiltration of the tumor to the surrounding tissues. FNAC is considered the most reliable method for the detection of malignancy in midline neck masses before surgical intervention [7]. As most lesions are cystic in nature, FNAC of the mural nodule under ultrasound guidance can improve the diagnostic accuracy by reducing the false negative results [8]. 

The treatment options for carcinoma of the thyroglossal duct cyst include cyst excision, Sistrunk operation, and Sistrunk operation along with total thyroidectomy. The Sistrunk operation involves resection of the thyroglossal cyst, the body of the hyoid bone, and the entire tract up to the foramen caecum at the base of the tongue. The 10 years survival rate of the patients undergoing simple cyst excision and Sistrunk operation is about 75% and 100% respectively [8]. Sistrunk procedure is now the standard surgical treatment for benign thyroglossal duct cysts as well as for malignancies with low-risk features. The high-risk features include patients above 45 years of age, prior history of radiation exposure, tumors larger than 1.5 cm, invasion of the cyst wall, tumor in the thyroid gland on imaging, and the presence of cervical lymphadenopathy [9]. Patients with these high-risk characteristics should undergo a total thyroidectomy along with a Sistrunk operation with or without cervical lymphadenectomy, depending on the involvement of cervical nodes. This should be followed by radioactive iodine ablation using Iodine-131 (^131^I) and thyroid-stimulating hormone (TSH) suppression thereafter.

The prognosis of papillary carcinoma arising in the thyroglossal duct cyst is excellent and squamous cell carcinomas have the worst prognosis. Long-term follow-up of these patients is mandatory as delayed metachronous papillary thyroid cancers have been reported arising in the thyroid gland several years after the initial surgery [10]. Follow-up consists of history and physical examination, sonography of the neck, and whole body scintigraphy.

## Conclusions

Malignancy should be suspected in elderly patients presenting with a thyroglossal cyst as the condition is very rare. A high-resolution ultrasonography or CT scan should be performed in all suspected malignant cases. FNAC under ultrasound guidance can help in sampling the mural nodule associated with the cystic lesions. Surgeons should be aware of this clinical condition as appropriate preoperative evaluation can influence the nature of surgical treatment in these patients.
